# Longitudinal validation of cognitive reserve proxy measures: a cohort study in a rural Chinese community

**DOI:** 10.1186/s13195-024-01451-6

**Published:** 2024-04-23

**Authors:** Hao Chen, Jin Hu, Shiqi Gui, Qiushuo Li, Jing Wang, Xing Yang, Jingyuan Yang

**Affiliations:** 1 Department of Epidemiology and Health Statistics, School of Public Health, The Key Laboratory of Environmental Pollution Monitoring and Disease Control, Guizhou Medical University, Guiyang, China; 2 School of Medicine and Health Management, Guizhou Medical University, Guiyang, China; 3 The Third People’s Hospital of Guizhou Province, Guiyang, China

**Keywords:** Cognitive reserve, Cognition, Confirmatory factor analyses, Measurement invariance

## Abstract

**Background:**

While evidence supports cognitive reserve (CR) in preserving cognitive function, longitudinal validation of CR proxies, including later-life factors, remains scarce. This study aims to validate CR’s stability over time and its relation to cognitive function in rural Chinese older adults.

**Methods:**

Within the project on the health status of rural older adults (HSRO), the survey included baseline assessment (2019) and follow-up assessment (2022). 792 older adults (mean age: 70.23 years) were followed up. The confirmatory factor analysis (CFA) was constructed using cognitive reserve proxies that included years of formal education, social support, hobbies, and exercise. We examined the longitudinal validity of the CR factor using confirmatory factor analyses and measurement invariance and explored the association of CR with cognition using Spearman’s correlation and Generalized Estimating Equations (GEE).

**Results:**

The results showed that CR’s CFA structure was stable over time (T0, χ^2^/*df*: 3.21/2; RMSEA: 0.02, and T1, χ^2^/*df*: 7.47/2; RMSEA: 0.05) and that it accepted both configural and metric invariance (Δχ^2^/*df* = 2.28/3, *P* = 0.52). In addition, it was found that CR had a stable positive relationship with cognitive function across time (T0, *r* = 0.54; T1, *r* = 0.49). Furthermore, longitudinal CR were associated with MMSE (*β* = 2.25; *95%CI* = 2.01 ~ 2.49).

**Conclusions:**

This study provided valuable evidence on the stability and validity of cognitive reserve proxy measures in rural Chinese older adults. Our findings suggested that cognitive reserve is associated with cognitive function over time and highlighted the importance of accumulating cognitive reserve in later life.

**Supplementary Information:**

The online version contains supplementary material available at 10.1186/s13195-024-01451-6.

## Introduction

As the world’s aging population continues to grow and dementia prevalence increases, the prevention and treatment of dementia have become a top priority for society worldwide [1, 2]. China, with its large aging population and high prevalence of dementia, faces an urgent need to address this issue [3, 4]. Although no treatment is available to slow or stop dementia, prevention of cognitive decline is an important strategy. Cognitive reserve (CR), as a fascinating concept, emphasizing the capacity of lifestyle choices and life events throughout one’s life to positively influence and enhance cognitive processes, thereby bolstering efficiency and flexibility in addressing cognitive decline [5]. Accumulated evidence indicated that CR could enhance cognitive adaptability and reduce sensitivity to brain aging, pathology, or injury, delaying clinical symptoms [5–7].

Nevertheless, since CR cannot be directly measured, it is generally operationalized using proxies such as education, occupation, physical exercise, and social activities [8, 9]. Even as numerous studies have shown an association between CR-related proxies and cognitive function, there is heterogeneity in the specific proxies used for CR assessment across different populations. These proxy factors may reflect the unique characteristics and contexts of the studied populations. For instance, studies have shown that education alone is associated with cognition in some populations, while other proxies such as leisure activities or occupation may not exhibit a significant relationship [10, 11]. This highlights the importance of considering population-specific factors when examining the relationship between CR-related proxies and cognitive function. Researchers found that higher childhood school performance and engagement in complex job environments during adulthood were associated with a reduced risk of dementia [12]. Another longitudinal study found that higher social support and engagement in leisure activities improve cognitive reserve in old age [13]. This underscores the importance of exploring CR-related proxies at different stages of life to understand their contributions to cognitive reserve [14]. The above studies imply that although education and occupation in early life are prerequisites for cognitive reserve in older adults, additional proxy indicators of cognitive reserve in later life may contribute to its enhancement, offering a new perspective for older adults facing declining cognition. However, it is crucial to exercise caution when interpreting changes in proxy measures, as cognitive reserve itself is not directly measurable. These proxy measures serve as indicators but may not fully capture the true changes in cognitive reserve. Therefore, establishing longitudinal measures to track the changes in proxy indicators of cognitive reserve over time and assessing their structural validity remain areas requiring further development. However, there has been little progress in establishing longitudinal measures of the change in proxy measures of CR over time and assessing structural validity [14–16].

Measurement invariance techniques are often used in the field of psychology to check the stability of latent measures across time, groups, and ethnicities [17]. It also is a way to enhance the fairness and validity of neurocognitive ability tests, and although this method is well established for use, it has not yet fully realized its potential in cognition [18]. According to the existing literature, these techniques have not yet been applied in studies of the CR model. In addition, recent studies have shown that older adults with dementia have lower levels of education and lower levels of occupational complexity as well [19], and rural older adults have worse CR and cognitive function compared to their urban counterparts [20]. In China, many rural older adults have low levels of education and have only worked in agriculture during their early life. Therefore, validating the longitudinal effectiveness of cognitive reserve in later life is crucial to confirm its value in delaying cognitive decline in this population. To address this knowledge gap, this study aims to investigate cognitive reserve proxy measures in older adults within a rural Chinese community, validate the structural stability of these measures over time, and estimate their relationship with cognitive function.

## Materials and methods

### Study design and participants

This is a cohort from the Guizhou rural older adults’ health study (HSRO) in China. The HSRO is a population-based prospective study conducted in Guizhou, China. The data were obtained using multistage cluster sampling; 12 villages were selected, and the baseline survey was conducted from July to August 2019. Participants were eligible if they were 60-year-old community volunteers who had lived in the area for at least 6 months. The study employed a two-wave (T0-T1) longitudinal survey design. This study included 1,654 older adults who were assessed for cognitive reserve-related proxy measures at baseline. In 2022 (T1), 792 individuals participated in the follow-up surveys. The study was approved by the Ethics Committee of Guizhou Medical University, and all the participants signed informed consent.

### Measurement

#### Cognitive reserve

(CR) is a theoretical framework that aims to understand the protective factors contributing to cognitive abilities in individuals. In our study, we collected data on four proxies of cognitive reserve: years of education, social support, hobbies, and exercise. To analyze these variables, we employed confirmatory factor analysis, which allowed us to construct a latent variable model representing cognitive reserve. This approach helps us examine the relationship between these proxies and their collective influence on cognitive abilities. Education was measured by 1 item; subjects reported the total number of years at school. The Social Support Rating Scale(SSRS), developed by Xiao [21], was used to measure the amount of social support. It had three dimensions (subjective support, objective support, and support utilization). There were 10 items in SSRS. And seven questions were answered on a four-point Likert scale, while the remaining questions were answered differently (calculating the number of support sources). Participants were asked a series of questions regarding their engagement in various hobbies and activities, including housework, outdoor activities (e.g., fishing, hiking), gardening, reading books and newspapers, raising poultry or livestock, playing cards or mahjong, watching TV and listening to the radio, participating in organized social activities (e.g., square dancing), as well as indicating if they had no hobbies or engaged in other hobbies not mentioned [22]. The questionnaire comprised ten items, including an item for indicating the absence of hobbies. The number of hobbies was calculated by assigning scores to the remaining items. The exercise component of the questionnaire was designed based on the common exercise durations of 30 and 60 min for the elderly population [23]. Participants were asked to indicate the amount of time they spent exercising each day using the following response options: (1) never, (2) 0–30 min, (3) 30–60 min, and (4) more than 60 min.

#### Cognition

The Chinese version of the Mini-Mental State Examination (MMSE) scale was used to evaluate individuals’ cognition [24, 25]. The test includes 11 items, and the scores can immediately reflect global cognition in clinical, research, and community settings. The scores range from 0 to 30. The changes in cognitive function observed during the follow-up period were categorized into two groups: one group with no reduction in cognitive function (Maintenance) and another group with a decline in cognitive function (Decline).

#### Covariates

The smoking category was divided into 3 categories: current smoking (defined as a total of > 100 cigarettes smoked in the past year), ever smoking (including quitting smoking for > 6 months) and never smoking. The alcohol consumption category was divided into 3 categories: regular drinking (defined as drinking on an average of ≥ 3–5 days per week in the past year) or ever/occasional drinking (defined as drink on an average of ≤ 1–2 days per week in the past year), and never drink. Participants in the study were asked which chronic diseases they were diagnosed with, and the number of chronic conditions was counted. Boxes are provided to ask participants if they have specific chronic diseases. These listed the chronic diseases included in the questionnaire, such as arthritis, hypertension, cardiovascular disease, stomach disease, cataracts, chronic lung disease, diabetes, asthma, reproductive disorders, and cancer. In addition, space was provided for participants to write down any other chronic diseases that were not listed.

### Statistical analysis

Frequency and median (Interquartile Range (IQR), or range) were used to describe demographic characteristics. Non-parametric tests were employed to analyze the data. The Wilcoxon’s signed rank test was utilized for within-group comparisons of continuous variables with repeated measures, such as comparing baseline and follow-up data within the same group. On the other hand, the Marginal Homogeneity (MH) test was used for longitudinal comparisons between different groups, examining the differences in data distributions across different time points.

To capture proxy factor data for cognitive reserve (CR) more accurately, we utilized continuous information as the preferred form. Confirmatory factor analysis (CFA) was conducted separately for the baseline and follow-up assessments to test model fit. CFA of the CR proxy factor structure evaluation produced eight indicators of goodness of fit: Chi square/df, Root Mean Square Error of Approximation (RMSEA), Comparative Fit Index (CFI), Tucker-Lewis Index (TLI), Normed Fit Index (NFI), Incremental Fit Index (IFI), Akaike information criterion (AIC), and Bayes information criterion (BIC). The following cut-off criteria for the fit index were used: (1) NFI > 0.90; (2) IFI > 0.90; (3) TLI > 0.90; (4) CFI > 0.90, and (5) RMSEA < 0.05; (6) Chi square/df < 5 [26]. For measurement invariance, a longitudinal two-group CFA was performed, testing for four increasingly stringent types of invariances: configuration, metric, scalar, and strict. Configural invariance was satisfied when indicator variables loaded onto the same factors across groups. Metric invariance is satisfied with adequate model fit when factor loadings remain constant across groups. Scalar invariance is satisfied when factor loadings and intercepts are held constant across groups when model fit is adequate. Strict invariance is satisfied when the factor loadings, intercepts, and residuals are constrained to be equal across groups when the model fit is adequate [27].

The factor scores for CR were obtained using the maximum likelihood method. Longitudinal changes in cognitive function were calculated using the formula ΔMMSE = MMSE_T1_ - MMSE_T0_, where MMSE_T1_ represents the follow-up Mini-Mental State Examination (MMSE) score and MMSE_T0_ represents the baseline MMSE score. Similarly, cognitive reserve was calculated as ΔCR = CR_T1_ - CR_T0_, where CR_T1_ represents the follow-up cognitive reserve score and CR_T0_ represents the baseline cognitive reserve score. Based on the above results, scores with the ΔMMSE greater than or equal to 0 were divided into the maintenance group, and those less than 0 were divided into the cognitive decline group. Similarly, the ΔCR was categorized into two groups: the group with increased CR (positive ΔCR) and the group with decreased CR (negative ΔCR). The Spearman’s correlation coefficients between CR scores and MMSE scores were calculated, and the Fisher Z method was used to estimate the significance of the difference between the longitudinal correlation coefficients. This test involves comparing the standard error of the difference between the two coefficients to the difference between the coefficients and calculating a Z-score. If the Z-score is larger than a critical value, the difference between the two coefficients is considered statistically significant [28]. Generalized estimating equations were employed to analyze the longitudinal relationship and interaction effects between cognitive reserve and cognition. The statistical analyses were performed using SPSS (IBM, Armonk, NY, USA, version 22.0) and R software (package: Lavaan, semTools, version 4.2.2).

## Results

A total of 792 study subjects entered the follow-up. The mean (SD) age at baseline assessment was 70.23 (5.87) years. There were 318 males and 474 females in the follow-up data; 96.7% of the subjects’ occupations were farmer only, and 81.2% had received less than six years of education (Table S1). At baseline and follow-up, there were statistically significant differences in the SSRS scores, the number of hobbies, the time spent being physically active, and the MMSE scores (Table 1).

**Table 1:** Longitudinal distribution of CR proxy factors, lifestyle, and number of chronic conditions

Characteristic	Baseline	Follow-up	Z /MH	P Value
	*n* = 792	*n* = 792		
SSRS, median (IQR)	39(10)	37(10)	-8.44	< 0.001
Exercise, hours, n (%)				
Never	417(52.7)	289(36.0)		
0~	151(19.1)	251(31.3)		
0.5 h~	131(16.5)	127(15.8)	-5.24	< 0.001
1 h~	93(11.7)	136(16.9)		
Hobbies, n (%)				
0~	711(89.8)	230(29.0)		
3~	78(9.8)	496(62.7)	-21.31	< 0.001
6~	3(0.4)	66(8.3)		
MMSE score, median (IQR)	22(8)	21(9)	-10.35	< 0.001
Smoking status, n (%)				
Never	523(66.0)	538(67.0)		
Ever	64(8.1)	58(7.2)	-0.79	0.43
Current	205(25.9)	207(25.8)		
Alcohol consumption, n (%)				
Never	515(65.0)	506(63.0)		
Ever/occasional	86(10.9)	94(11.7)	1.40	0.16
Regular	191(24.1)	203(25.3)		
Number of chronic conditions, median (range)	1(0,8)	1(0,6)	-7.22	< 0.01

*Note* SSRS, Social Support Rating Scale; MMSE, Mini-Mental State Examination

The CR latent variables constituted by the CFA are shown in Figure S1; the highest factor loadings were for hobbies (0.69) at baseline and for social support (0.47) at follow-up. There were excellent goodness-of-fit results at T0 (χ^2^/*df*: 3.21/2; RMSEA: 0.02; CFI: 0.99; TLI: 1.00; NFI: 0.99; IFI: 1.00) and T1 (χ^2^/*df*: 7.47/2; RMSEA: 0.05; CFI: 0.96; TLI: 0.87; NFI: 0.94; IFI: 0.96) as presented in Table S2. By adding constraints (Table 2; Tables S3-S6), the model passes only configuration invariance and metric invariance (Δ (Metric – Configural model): Δχ^2^ = 2.28; Δ*df* = 3; ΔRMSEA= -0.012; ΔCFI = 0.003; ΔTLI = 0.028).

**Table 2:** Longitudinal measurement invariance of the CR model

Model	χ^2^	df	RMSEA	CFI	TLI	AIC	BIC	P
Configural Invariance	9.165	4	0.040	0.981	0.944	28388.806	28517.631	
Metric Invariance	11.451	7	0.028	0.984	0.973	28385.092	28497.814	
Scalar Invariance	97.637	9	0.112	0.681	0.575	28467.278	28569.264	
Strict Invariance	106.94	13	0.096	0.662	0.688	28468.581	28549.097	
Δ(metric – config)	2.2861	3	-0.012	0.003	0.028	-3.714	-19.817	0.52*
Δ(scalar – metric)	86.186	2	0.083	-0.303	-0.398	82.186	71.451	< 0.001
Δ(strict – scalar)	9.303	4	-0.016	-0.019	0.113	1.303	-20.167	0.053

*Notes* AIC = Akaike’s Information Criterion; BIC = Bayesian Information Criteria; CFI = comparative fit index; RMSEA = root mean square error of approximation; TLI = Tucker–Lewis index; * indicated an accepted model

**Fig. 1:**
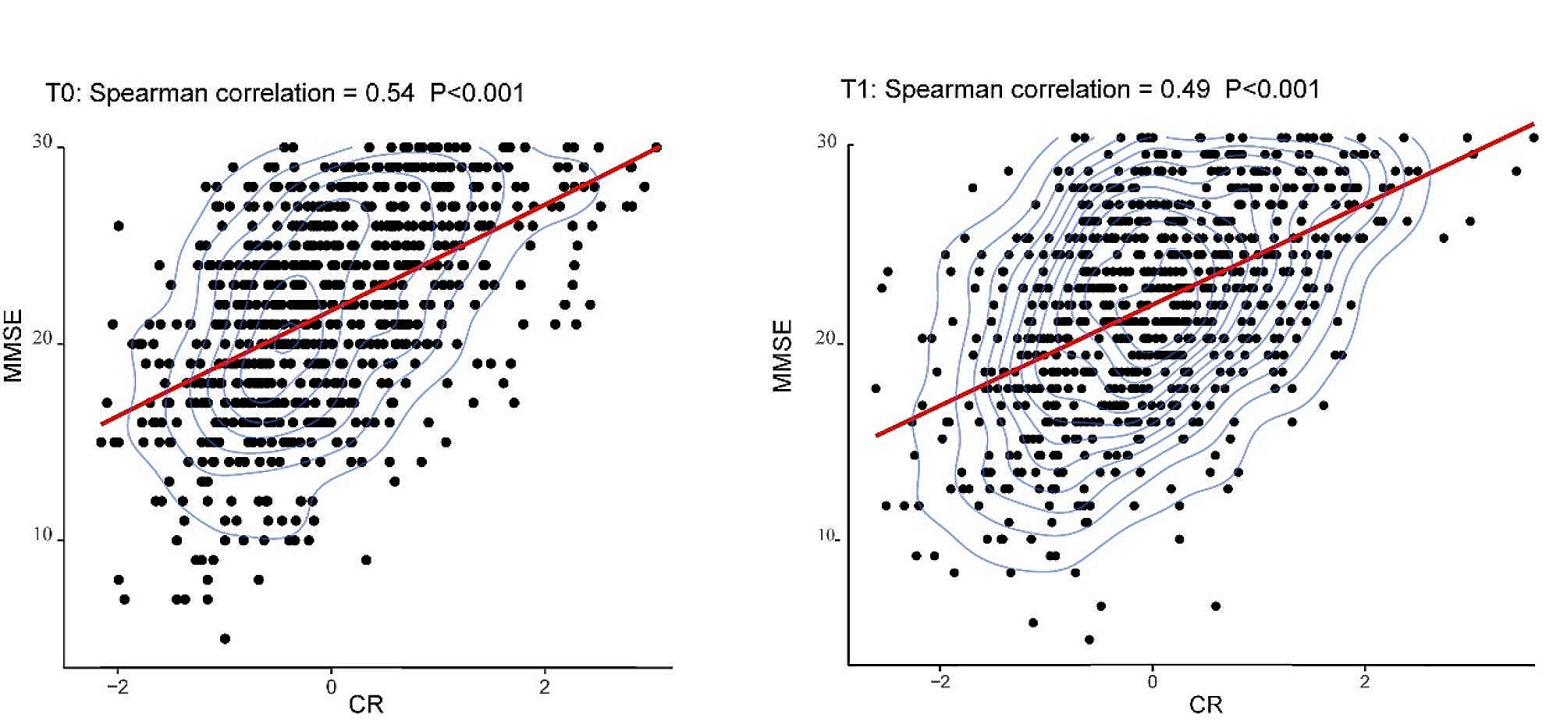
Correlation of CR with MMSE scores in two waves of study. *Note*: T0 for baseline, T1 for follow-up

**Fig. 2:**
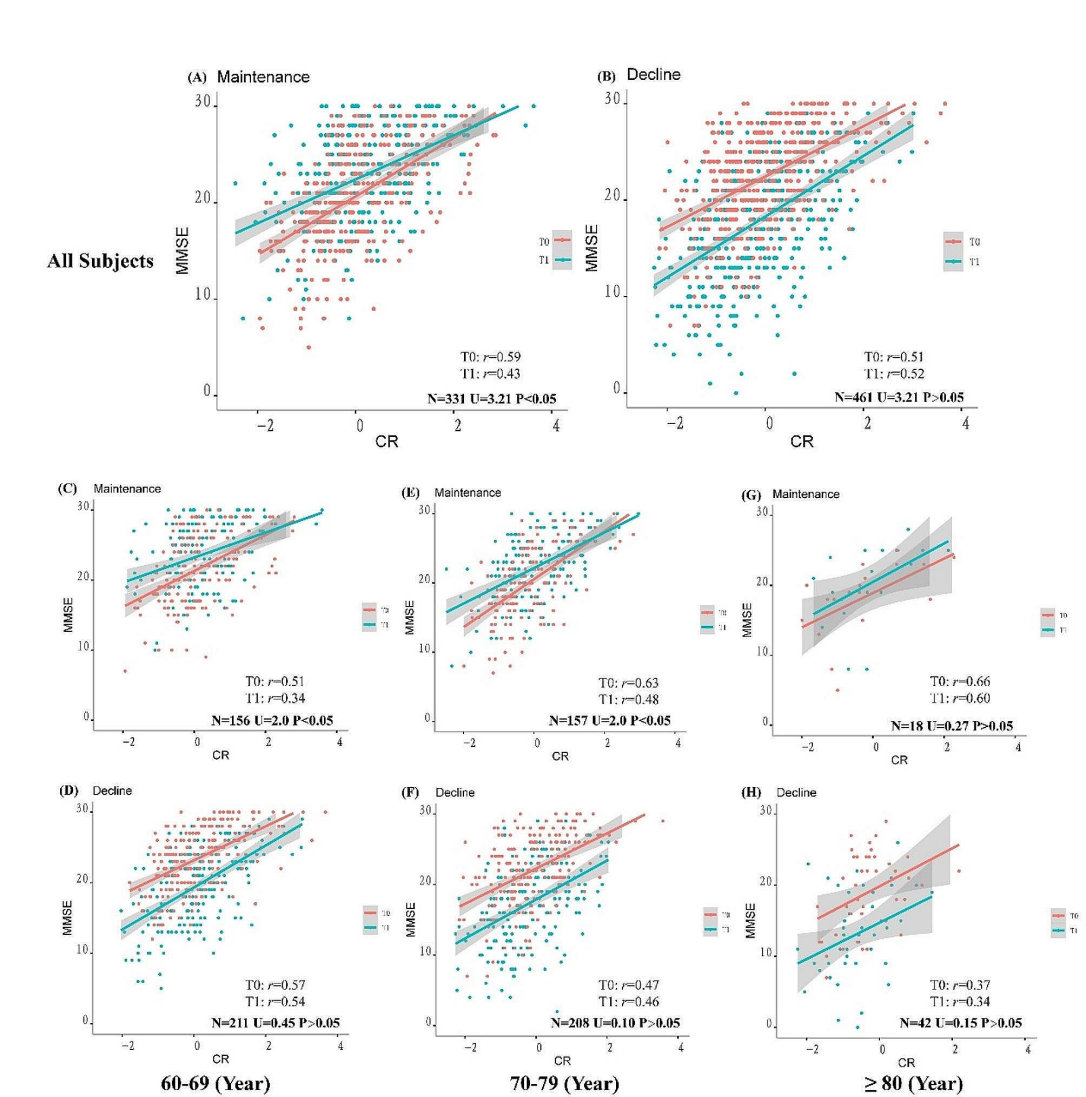
Comparison of longitudinal correlation coefficients between CR and MMSE in different cognitive groups. *Note*: (**A**) and (**B**) show the longitudinal comparison of the Z-transformed correlation coefficients of the cognitive maintenance group and the decline group for all subjects; (**C**), (**D**) are longitudinal comparisons for the 60–69 age group; (**E**), (**F**) are longitudinal comparisons for the 70–79 age group; (**G**), (**H**) are longitudinal comparisons for the ≥80 age group. T0 for baseline, T1 for follow-up

CR model factor scores were significantly positively correlated with cognitive function, either at baseline or follow-up (Fig. 1). The cognitive maintenance group exhibited a higher positive ΔCR compared to the cognitive decline group (Figure S2). When the longitudinal changes in the correlation coefficient between MMSE and cognitive reserve (CR) were examined, no significant difference in the correlation coefficient was seen for either the increased or decreased CR groups (Figure S3). Similarly, in Fig. 2(A, B), no significant longitudinal changes in correlation coefficients were identified in the cognitive decline group. However, in the cognitive maintenance group, a statistically significant difference in the longitudinal correlation coefficient between MMSE and CR was detected (*P* < 0.05). Further age stratification (referenced to baseline age) showed that the correlations between CR and MMSE scores over time were statistically different for subjects ages 60–69 (*N* = 156; T0: *r* = 0.51; T1: *r* = 0.35) and 70–79 (*N* = 157; T0: *r* = 0.63; T1: *r* = 0.48) in the cognitive maintenance group (Fig. 2). Generalized estimating equations revealed longitudinal associations between CR and cognitive functioning. Further analyses indicated that the relationships between CR and MMSE scores differed significantly across cognitive subgroups. Interactions were also observed with both sex and age (Table 3).

**Table 3:** Longitudinal association of cognition with CR

Variable	β	SE	95%CI	P-value
CR	2.25	0.12	2.01 ~ 2.49	< 0.001
Age group	-1.42	0.19	-1.79~-1.05	< 0.001
SEX	-2.58	0.25	-3.07~-2.09	< 0.001
Time (T0 VS T1)	-1.72	0.23	-2.16~-1.27	< 0.001
CR group (Decreased VS Increased)	0.06	0.23	-0.39 ~ 0.52	0.79
CF group (Decline VS Maintenance)	1.11	0.23	0.66 ~ 1.56	< 0.001
**Interaction**				
CR*Time	0.31	0.23	-0.14 ~ 0.76	0.17
CR*Time*CF group	1.11	0.42	0.28 ~ 1.94	0.01
CR*Age group	-0.46	0.19	-0.83~-0.09	0.01
CR*SEX	0.54	0.24	0.06 ~ 1.02	0.03

*Notes* CR, cognitive reserve; CF, cognitive function; T0, T1 indicate baseline or follow-up

## Discussion

This study assessed the longitudinal stability and validity of proxy indicators of cognitive reserve in a rural Chinese community. Building upon Cognitive Reserve (CR) theory, our study identified a set of CR proxies. The Confirmatory Factor Analysis (CFA) model demonstrated a high fit at two separate time points, and the longitudinal structure confirmed the configuration and metric invariance of measurement. Subsequent analysis revealed robust positive correlations between the CR model’s factor scores and cognitive function. Further analysis showed that the factor scores of the CR model were robustly positively correlated with cognitive function. As far as we know, this is the first study to focus on measurement invariance for the longitudinal validation of the assessment CR model.

In recent years, Kartschmit et al. [14] summarized the shortcomings of currently available CR assessment tools and concluded that it was necessary to extend the investigation to different populations due to their different experiences in terms of CR proxy parameters. People from various cultural and lifestyle backgrounds may have a diverse variety of proxy parameters to improve the CR. This study conducted on older adults in rural communities in China, most of whom have low education and have been farmers all their lives. They may also have the assumption that certain exposures relatively late in life can also contribute to CR. Similarly, studies have shown that occupation is not associated with cognition in subjects with low levels of education in developing countries [11]. The present investigation found significant high levels of CFA fit indices at baseline and at follow-up. A Healthy Brain Project cohort found consistent results, demonstrating the stability of the longitudinal structure of the CFA in CR [16]. Moreover, this study applied measurement invariance, aiming to ensure reliable conclusions about real CR changes across time. According to measurement invariance conventions and reporting, this study accepted both configural invariance and metric invariance. However, while full invariance is preferred, it may not always be achieved. This partial invariance could be attributed to various factors, such as changes in the sample composition or modifications in the measures employed [29]. Similarly, some cognitive-related studies have failed to meet the most stringent invariance steps, finding significant changes in intercepts and residuals over time [30]. These differences may be attributed to sample characteristics, and there may indeed be real differences across time in the CR model.

Consistent with previous longitudinal studies of CR [31, 32], this study supported the theory of the CR model that CR-related proxies were positively associated with cognitive function at either baseline or follow-up. Furthermore, our findings indicated that older adults in the cognitive maintenance group demonstrated a higher ΔCR, suggesting that the long-term accumulation of cognitive reserve may contribute to the preservation of cognitive performance at a stable level. This aligns with previous research suggesting that the maintenance of cognitive function is associated with cognitive reserve [33]. In the cognitive maintenance group, our findings revealed a notable decrease in the correlation coefficients between CR and MMSE scores over time, including different age groups. This intriguing observation may be aligned with the notion put forth by Montine et al. [34], suggesting that cognitive reserve “consumption” is manifested in cognitive performance prior to the onset of cognitive decline. Thus, the observed decline in correlation coefficients may indicate the utilization or “consumption” of cognitive reserve resources in maintaining cognitive performance at a stable level. Conversely, in the cognitive decline group, we observed no significant changes in the correlation coefficients between CR and MMSE scores over time. This finding suggests that, in the context of age-related cognitive decline, cognitive networks may undergo complex and dynamic processes involving the recruitment of additional neural resources for compensation. This observation aligns with the hypothesis proposed by Cabeza et al. [35] and implies that maintenance and compensation mechanisms could potentially occur simultaneously. Notably, the GEE model results similarly demonstrated statistically significant differences in the associations between CR and MMSE across cognitive subgroups, with interactions observed for both age groups and sexes. However, further research is needed to fully elucidate the intricate dynamics of these processes and their impact on the association between cognitive reserve and cognitive function. It is important to acknowledge that the concepts surrounding cognitive reserve remain subjects of ongoing debate and warrant further investigation through longitudinal studies. Furthermore, our study uncovered stable correlation coefficients between CR and MMSE scores in both the groups with increased and decreased CR over time. These intriguing findings suggest that changes in CR accumulation over time may not significantly impact the association with cognitive function. In other words, our results do not support the assumption that a greater accumulation of cognitive reserve necessarily translates to a stronger correlation with cognitive abilities. However, in the CR increased group, the intercept difference in cognitive level at different times was large, compared to the CR decreased group. Whether this is consistent with the existing evidence finding a more rapid rate of exacerbated cognitive decline in subjects with higher reserves requires further follow-up [36].

In terms of factor loading in longitudinal CFA, social support and hobbies have better factor loading than physical activity across time. Consistent with a cross-sectional study by the China Health and Retirement Project, older adults participating in hobby groups have better cognitive performance [37]. The low factor loading of physical activity in the reserve model may be related to the fact that older people in rural China spend more than half of each day with sedentary behavior [38]. While longer daily physical exercise would be expected to have positive effects on cognitive resilience, the influence of sedentary behavior over a significant portion of the day could potentially attenuate these effects. The majority of our participants, although having a background in farming, are not currently engaged in active agricultural work. This demographic shift from active farming to less physically demanding daily activities may contribute to the observed sedentary lifestyle, which is consistent with the lower factor loading of physical activity in our cognitive reserve model. Social support’s higher factor loading compared to physical activity likely results from rural communities’ strong social bonds, providing a steadier and more significant boost to cognitive reserve than inconsistent physical activity in individuals moving away from labor-intensive work. However, these findings should be interpreted with caution, acknowledging the need for further research to unravel the complex interplay between sedentary behavior, cognitive reserve, and the context of rural Chinese older adults. Additionally, the possibility of reverse causation, where cognitive decline might lead to reduced physical and social activities, calls for more in-depth investigation in future studies to clarify these intricate relationships.

A noteworthy strength of our study utilization of latent variables allows capturing more current CR-related factors each time to reduce recall bias and to ensure that CR measurements are valid across life stages. Nevertheless, some limitations of our study should be noted. Firstly, while the cognitive reserve (CR) proxies used in our study are commonly utilized indicators, they may not fully capture the CR in our specific population of rural older adults. This could potentially limit the generalizability of our findings to other populations or settings. Secondly, the small sample size of very old older adults and the limited number of follow-up waves may have affected the statistical power of our analysis and hindered our ability to capture the dynamic changes in CR over time. It is important to acknowledge that a larger sample size and a longer follow-up period would provide a more robust assessment of the relationship between CR and cognitive outcomes. Thirdly, the measurement of cognitive function using tools like the Mini-Mental State Examination (MMSE) is subject to measurement errors and may have ceiling effects, particularly in populations with high baseline cognitive performance. This could limit our ability to detect subtle changes in cognitive performance and affect the accuracy of the observed correlations. Lastly, the absence of cognitive-related biological indicators, such as neuroimaging or biomarkers, and the limited scope of cognitive status measures used in our study may have restricted our comprehensive assessment of cognitive reserve and its association with cognitive function.

In conclusion, this study provided confirmatory evidence of the longitudinal stability and validity of proxy indicators of cognitive reserve in low-educated rural older adults and indicated that cognitive reserve factors correlate with cognitive performance. Our results highlight the importance of proxy variables for late-life CR throughout the lifespan in preserving cognitive function. They play a crucial role in promoting healthy aging among rural Chinese older adults.

### Electronic supplementary material

Below is the link to the electronic supplementary material.

Supplementary Material 1

## Data Availability

The datasets that support the findings of this study are available on request from the corresponding author (Jingyuan Yang, e-mail: yang8880@sina.com). The data is not publicly available due to privacy or ethical restrictions.
