# Supplementary material for: Longitudinal validation of cognitive reserve proxy measures: a cohort study in a rural Chinese community
Source: Alzheimers Res Ther. 2024 Apr 23;16:87. doi: 10.1186/s13195-024-01451-6 (PMC11036581; doi:10.1186/s13195-024-01451-6)
Supplement: Supplementary file 1 — Supplementary Material 1 [file 13195_2024_1451_MOESM1_ESM.docx]

**Longitudinal validation of cognitive reserve proxy measures: a cohort study in a rural Chinese community**

(Supplemental files)

Table S1 Distribution of socio-demographic information

| Characteristic | Baseline | Follow-up |
| --- | --- | --- |
|  | n=1654 | n=792 |
| Age, Y, mean (SD), | 71.36(6.57) | 73.62(5.84) |
| Male/Female, | 690/954 | 318/474 |
| Farmer, n (%) | 1510(91.3) | 766(96.7) |
| Education level, Y, n (%) |  |  |
| 0~ | 991(59.9) | 448(56.6) |
| 3~ | 303(18.3) | 195(24.6) |
| 6~ | 222(13.4) | 107(13.5) |
| 9~ | 138(8.3) | 42(5.3) |
| SSRS, median (IQR) | 38(9) | 37(10) |
| Exercise, hours, n (%) |  |  |
| Never | 928(56.1) | 289(36.0) |
| 0~ | 295(17.8) | 251(31.3) |
| 0.5h~ | 246(14.9) | 127(15.8) |
| 1h~ | 185(11.2) | 136(16.9) |
| Hobbies, n (%) |  |  |
| 0~ | 1496(90.4) | 230(29.0) |
| 3~ | 152(9.2) | 496(62.7) |
| 6~ | 6(0.4) | 66(8.4) |
| MMSE score, median (IQR) | 21(8) | 21(9) |
| Smoking status, n (%) |  |  |
| Never | 1068(64.6) | 538(67.0) |
| Ever | 136(8.2) | 58(7.2) |
| Current | 450(27.2) | 207(25.8) |
| Alcohol consumption, n (%) |  |  |
| Never | 1042(63.0) | 506(63.0) |
| Ever/occasional | 174(10.5) | 94(11.7) |
| Regular | 438(26.5) | 203(25.3) |
| Chronic conditions, n (%) |  |  |
| Yes | 1039(62.8) | 460(58.1) |
| No | 615(37.2) | 332(41.9) |

Note: SSRS, Social Support Rating Scale; MMSE, Mini-Mental State Examination.

Table S2 Goodness-of-Fit Statistics for T0 and T1 Confirmatory Factor Analysis Models

| Goodness-of-fit | T0 | T1 |
| --- | --- | --- |
| *χ^2^/df* | 3.21/2 | 7.47/2 |
| Root mean square error of approximation (RMSEA) | 0.02 | 0.05 |
| Comparative Fit Index (CFI) | 0.99 | 0.96 |
| Tucker-Lewis Index (TLI) | 1.00 | 0.87 |
| Bentler-Bonett Normed Fit Index (NFI) | 0.99 | 0.94 |
| Bollen's Incremental Fit Index (IFI) | 1.00 | 0.96 |
| Akaike’s Information Criterion (AIC) | 24395 | 14662 |
| Bayesian Information Criteria (BIC) | 24460 | 14719 |

Table S3 Descriptive information of the cognitive reserve proxies[n=792]

| CR proxies |  | Baseline(T0) |  |  | Followup(T1) |
| --- | --- | --- | --- | --- | --- |
|  | Mean | SD |  | Mean | SD |
| Yoe | 1.00 | 2.59 |  | 1.00 | 2.59 |
| SSRS | 38.18 | 7.36 |  | 36.35 | 7.71 |
| Hobbies | 1.31 | 0.95 |  | 3.31 | 1.57 |
| Exercrise | 1.81 | 1.06 |  | 2.13 | 1.09 |

Note: Yoe, Years of education; SSRS, Social Support Rating Scale; SD = standard deviation.

Table S4 Unconstrained unstandardized factor loadings, intercepts, and variances in the configural invariance model of the CR proxies over two waves

| CR proxies |  | Baseline(T0) |  |  |  | Followup(T1) |  |
| --- | --- | --- | --- | --- | --- | --- | --- |
|  | Factor loadings | Intercepts | Variances |  | Factor loadings | Intercepts | Variances |
| Yoe | 1.00 | 2.59 | 6.78 |  | 1.00 | 2.59 | 6.93 |
| SSRS | 2.08 | 38.94 | 45.22 |  | 2.95 | 36.35 | 46.26 |
| Hobbies | 0.52 | 1.38 | 0.48 |  | 0.57 | 3.31 | 1.97 |
| Exercrise | 0.19 | 1.87 | 1.09 |  | 0.28 | 2.13 | 1.06 |

Note: Yoe, Years of education; SSRS, Social support Rating Scale; All parameters are significant at p < 0.05 for the two-tailed test.

Table S5 Constrained unstandardized factor loadings and unconstrained intercepts and variances in the metric invariance model of the CR proxies over two waves

| CR proxies |  | Baseline(T0) |  |  |  | Followup(T1) |  |
| --- | --- | --- | --- | --- | --- | --- | --- |
|  | Factor loadings | Intercepts | Variances |  | Factor loadings | Intercepts | Variances |
| Yoe | 1.00 | 2.59 | 6.86 |  | - | 2.59 | 6.64 |
| SSRS | 2.33 | 38.94 | 44.69 |  | - | 36.35 | 48.57 |
| Hobbies | 0.54 | 1.38 | 0.49 |  | - | 3.31 | 1.93 |
| Exercrise | 0.21 | 1.87 | 1.49 |  | - | 2.13 | 1.08 |

Note: Yoe, Years of education; SSRS, Social support Rating Scale; All parameters are significant at p < 0.05 for the two-tailed test.

Table S6 Constrained unstandardized factor loadings, intercepts, and unconstrained variances in the scalar invariance model of the CR proxies over two waves

| CR proxies |  | Baseline(T0) |  |  |  | Followup(T1) |  |
| --- | --- | --- | --- | --- | --- | --- | --- |
|  | Factor loadings | Intercepts | Variances |  | Factor loadings | Intercepts | Variances |
| Yoe | 1.00 | 2.89 | 8.10 |  | - | - | 8.13 |
| SSRS | 2.69 | 40.52 | 51.47 |  | - | - | 56.88 |
| Hobbies | 0.54 | 3.31 | -1.10 |  | - | - | -0.04 |
| Exercrise | 0.21 | 2.09 | 1.14 |  | - | - | 1.17 |

Note: Yoe, Years of education; SSRS, Social support Rating Scale; All parameters are significant at p < 0.05 for the two-tailed test.


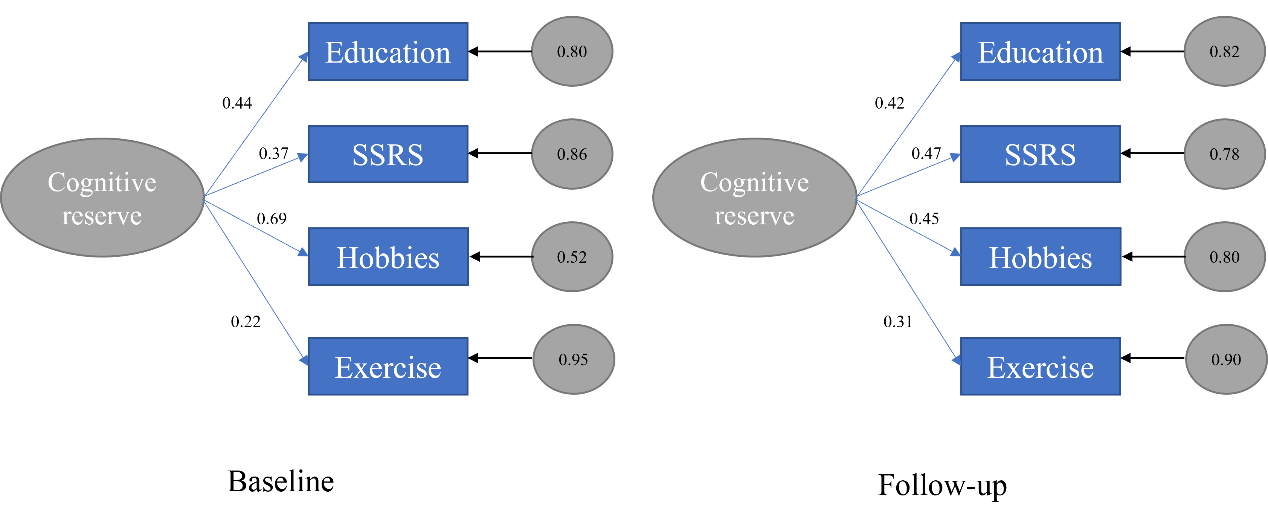


Figure S1 Confirmatory factor analysis of CR model


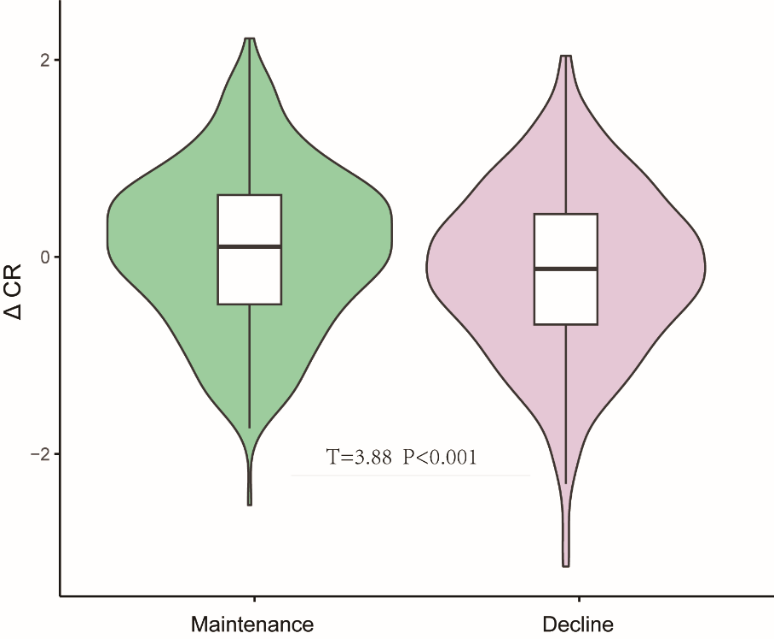


Figure S2 Distribution of CR changes in different cognitive groups


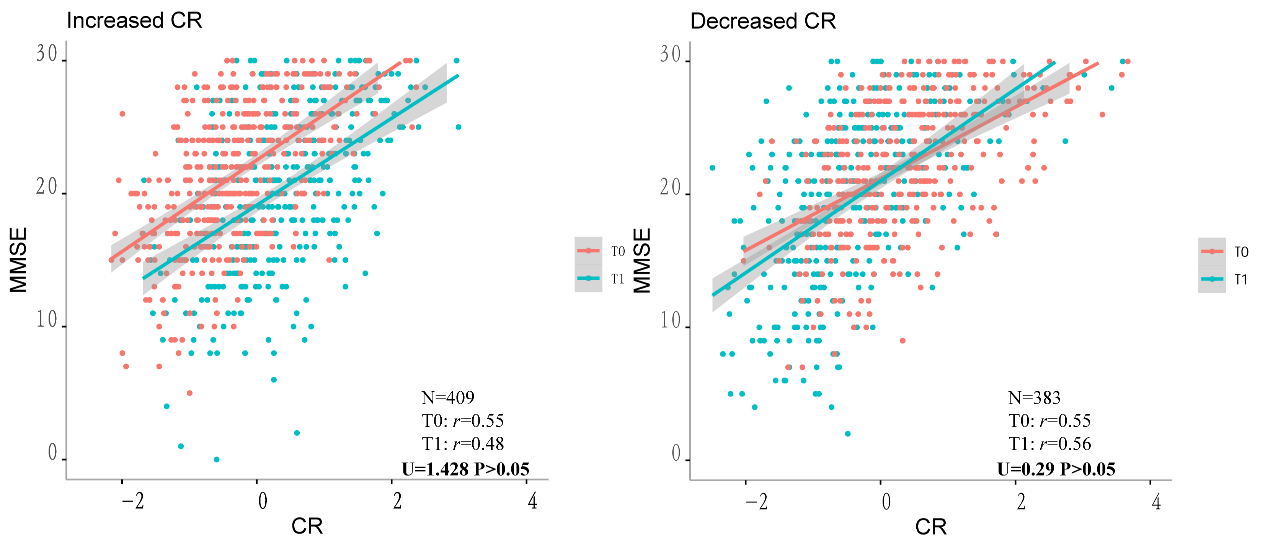


Note: T0 for baseline, T1 for follow-up.

Figure S3 Comparison of longitudinal correlation coefficients between CR and MMSE in different CR groups
